# Low Molecular Weight Dextran Sulfate (ILB^®^) Administration Restores Brain Energy Metabolism Following Severe Traumatic Brain Injury in the Rat

**DOI:** 10.3390/antiox9090850

**Published:** 2020-09-10

**Authors:** Giacomo Lazzarino, Angela Maria Amorini, Nicholas M. Barnes, Lars Bruce, Alvaro Mordente, Giuseppe Lazzarino, Valentina Di Pietro, Barbara Tavazzi, Antonio Belli, Ann Logan

**Affiliations:** 1 UniCamillus-Saint Camillus International University of Health Sciences, Via di Sant’Alessandro 8, 00131 Rome, Italy; giacomo.lazzarino@unicamillus.org; 2 Department of Biomedical and Biotechnological Sciences, Division of Medical Biochemistry, University of Catania, Via S. Sofia 97, 95123 Catania, Italy; amorini@unict.it; 3 National Institute for Health Research Surgical Reconstruction and Microbiology Research Centre, Queen Elizabeth Hospital, Edgbaston, Birmingham B15 2TH, UK; n.m.barnes@bham.ac.uk (N.M.B.); a.belli@bham.ac.uk (A.B.); 4 Institute of Clinical Sciences, College of Medical and Dental Sciences, University of Birmingham, Edgbaston, Birmingham B15 2TT, UK; 5 Tikomed AB, 26303 Viken, Sweden; lars.bruce@tikomed.com; 6 Department of Basic Biotechnological Sciences, Intensive and Perioperative Clinics, Catholic University of the Sacred Heart of Rome, Largo F. Vito 1, 00168 Rome, Italy; alvaro.mordente@unicatt.it; 7 Department of Laboratory Sciences and Infectious Disease, Fondazione Policlinico Universitario A. Gemelli IRCCS, Largo A. Gemelli 8, 00168 Rome, Italy; 8 Neurotrauma and Ophthalmology Research Group, Institute of Inflammation and Ageing, College of Medical and Dental Sciences, University of Birmingham, Edgbaston, Birmingham B15 2TT, UK; 9 Axolotl Consulting Ltd., Droitwich, Worcestershire WR9 0JS, UK

**Keywords:** severe traumatic brain injury, low molecular weight dextran sulfate, mitochondrial dysfunction, energy metabolism, *N*-acetylaspartate, nicotinic coenzymes, oxidative/nitrosative stress, ascorbate, reduced glutathione (GSH), HPLC

## Abstract

Traumatic brain injury (TBI) is the leading cause of death and disability in people less than 40 years of age in Western countries. Currently, there are no satisfying pharmacological treatments for TBI patients. In this study, we subjected rats to severe TBI (sTBI), testing the effects of a single subcutaneous administration, 30 min post-impact, of a new low molecular weight dextran sulfate, named ILB^®^, at three different dose levels (1, 5, and 15 mg/kg body weight). A group of control sham-operated animals and one of untreated sTBI rats were used for comparison (each group *n* = 12). On day 2 or 7 post-sTBI animals were sacrificed and the simultaneous HPLC analysis of energy metabolites, *N*-acetylaspartate (NAA), oxidized and reduced nicotinic coenzymes, water-soluble antioxidants, and biomarkers of oxidative/nitrosative stress was carried out on deproteinized cerebral homogenates. Compared to untreated sTBI rats, ILB^®^ improved energy metabolism by increasing ATP, ATP/ adenosine diphosphate ratio (ATP/ADP ratio), and triphosphate nucleosides, dose-dependently increased NAA concentrations, protected nicotinic coenzyme levels and their oxidized over reduced ratios, prevented depletion of ascorbate and reduced glutathione (GSH), and decreased oxidative (malondialdehyde formation) and nitrosative stress (nitrite + nitrate production). Although needing further experiments, these data provide the first evidence that a single post-injury injection of a new low molecular weight dextran sulfate (ILB^®^) has beneficial effects on sTBI metabolic damages. Due to the absence of adverse effects in humans, ILB^®^ represents a promising therapeutic agent for the treatment of sTBI patients.

## 1. Introduction

Traumatic brain injury (TBI) is one of the most common acute neurodegenerative diseases and represents the leading cause of death for people less than 45 years of age in Western countries. The incidence of TBI is on the rise, and by 2020 the World Health Organization estimates it will be the largest cause of disability worldwide [1]. Depending on the severity of the symptoms related to TBI (evaluated by the Glasgow Coma Scale), TBI may be classified as mild (mTBI), moderate, or severe (sTBI). Severe TBI is characterized by a high mortality rate and those who survive often suffer from profound disabilities with permanent impairment of cognitive, physical, and psychosocial functions, associated with a diminished or altered state of consciousness and inability to be independent, work correctly, and maintain social relationships [2].

To date, there are no satisfying pharmacological treatments capable of decreasing mortality/morbidity and improving recovery of sTBI patients [2,3]. Clinical and therapeutic management are complicated by the inhomogeneity of head injured patients, including the diversity of adverse consequences associated with subdural hematomas, subarachnoid hemorrhages, cerebral hypoxia from respiratory difficulties, and the invasive neurosurgical interventions used to control cerebral perfusion pressure [4,5,6]. Damage associated with any of the aforementioned complications may augment damage caused by sTBI, concomitantly requiring additional specific treatments and decreasing the effectiveness of TBI targeted therapies. An additional, but no less important, problem contributing to the lack of effective pharmacological therapies for sTBI patients is the highly variable time elapsed between TBI occurrence and arrival in Emergency Departments for initiation of patients’ treatment [7].

Even with the absence of the aforementioned variables, TBI is a complicated pathology in which the inevitable primary insult (the impact force acting on the brain tissue) is directly responsible for the induction of a secondary insult. This is characterized by a rapid cascade of biochemical, metabolic, and molecular changes causing profound modifications of various cerebral cell functions [8,9], often ultimately leading to cell necrosis and apoptosis with significant neuronal loss [10,11]. The severity of this cellular and molecular damage depends on the impact force acting on the cerebral tissue [12]. Mitochondrial dysfunction occurs in the post-injured brain [13,14], causing an imbalance of ATP production and consumption, with consequent energy crisis [15]. This triggers the intrinsic apoptotic pathway [16], and increasing levels of reactive oxygen species (ROS) and reactive nitrogen species (RNS) with associated decreased levels of cell antioxidants [17] causing an insurgence of oxidative/nitrosative stress [18]. Particularly this last phenomenon is responsible for the activation of microglia leading to a generalized inflammatory response with the balance shifted to production of proinflammatory cytokines [19,20,21].

In experimental graded TBI, we previously demonstrated a correlation between the severity of TBI and the energy deficit associated with an increased rate of anaerobic metabolism [22] and mitochondrial dysfunction [23,24], thereby initiating tissue damage mediated by reactive ROS and RNS [25,26]. Moreover, we identified *N*-acetylated amino acid *N*-acetylaspartate (NAA) as a reliable surrogate biomarker to monitor the state of the energetic metabolism in vivo [27,28]. Indeed, as mitochondrial NAA biosynthesis has a high indirect energy expenditure, changes in NAA intracerebral concentrations are closely related to changes of parameters related to energy metabolism, such as ATP, guanosine triphosphate (GTP), adenosine diphosphate (ADP), adenosine monophosphate (AMP), Acetyl-CoA, CoA-SH, and oxidized nicotinamide adenine dinucleotide (NAD^+^), and to mitochondrial phosphorylating capacity (ATP/ADP) [29,30].

The low molecular weight dextran sulfate (LMW-DS) used in this study (ILB^®^) is a branched polysaccharide with an average molecular weight of 5 kDa and contains molecules spanning approximately 3–8 kDa, in which the α-d-glucopyranose-2,4-sulfate monomers are linked, in 95% of cases, by α-1,6-glycosidic bonds (the main linear chain) and, in the remaining 5% of cases, by α-1,3-glycosidic bonds (the branches) (see patent publication WO 2016/076780—New dextran sulfate). In ILB^®^, sulfur represents ~17% of its total mass. While high molecular weight dextran sulfate possesses toxic effects, inducing cancer development and inflammation [31,32,33], ILB^®^ is a proven safe and well-tolerated LMW-DS. Previous studies showed that LMW-DS dose-dependently limits the instant blood-mediated inflammatory reaction (IBMIR) [34,35], inhibits E-selectin-mediated adhesion of neutrophils to endothelial cells [36,37], prevents adherence of peritoneal metastases [38], and improves the effectiveness of therapy and reduces mortality in patients with acute cerebral infarction treated with the thrombolytic agent urokinase [39]. Of particular mechanistic relevance to TBI, subcutaneous injection of ILB^®^ induces a rapid release of pharmacologically relevant levels of Hepatocyte Growth Factor (HGF) into the circulation in laboratory animals and healthy human volunteers, which may provide a neurotrophic stimulus to the injured central nervous system (CNS) [35].

In the present study, using the weight drop impact acceleration model of closed-head trauma [40], we assessed the dose-response effects of ILB^®^, subcutaneously administered 30 min after injury, on mitochondrial functions, energy metabolism, and oxidative/nitrosative stress of the brain tissue of rats experiencing sTBI, by evaluating concentrations of high energy phosphates, oxidized and reduced nicotinic coenzymes, *N*-acetyl aspartate (NAA), ascorbate, reduced glutathione (GSH), malondialdehyde (MDA), nitrite and nitrate, oxypurines (hypoxanthine, xanthine, and uric acid), and nucleosides (inosine, guanosine, and adenosine).

## 2. Materials and Methods

### 2.1. Induction of sTBI, Drug Dosage and Mode of Administration, and Drug Administration Protocol

The experimental study was approved by the Ethical Committee of the Catholic University of the Sacred Heart of Rome, Italy (approval 1F295.52, released on 10/20/2017), and by the Ethical Committee of the Italian Ministry of Health (approval No. 78/2018-PR, released on 02/05/2018).

Male Wistar rats (*n* = 160) of 300–350 g body weight were used. They were fed a standard laboratory diet and water ad libitum in a controlled environment. As the accepted anesthetic mixture, animals received 35 mg/kg body weight (b.w.) ketamine and 0.25 mg/kg b.w. midazolam by intramuscular injection. Diffuse severe TBI (sTBI) was induced according to the “weight drop” impact acceleration model of diffuse TBI set up by Marmarou A. et al. [40]. Severe TBI was induced by dropping a 450 g weight from 2 m height onto the rat head protected by a helmet (metal disk previously fixed on the skull using dental cement) in order to uniformly distribute the mechanical force to the brain. Rats were placed prone on a bed of specific polyurethane foam inserted in a special container; this foam dissipates the major part of the potential energy (deriving from the mechanical forces) and prevents any rebound of the animal after the impact that could produce spinal damages.

Animals suffering from skull fracture, seizures, nasal bleeding, or that did not survive the impact (4/52, with a mortality rate of the 7.7%) were excluded from the study. All animals surviving the impact survived for the entire observational period, up to 7 days after TBI. After 2 or 7 days from sTBI induction, rats were anesthetized again and immediately sacrificed. According to our previous studies [22,23,24,25,26,27,28,29,30], these time points are coincident with deep biochemical, metabolic, and molecular derangement (2 days) or with a recovery (7 days) of nervous cell functions.

Animals killed at both 2 and 7 days post-injury were divided into four groups, with 12 rats in each: (1) sTBI only; (2) sTBI + 1 mg/kg b.w. ILB^®^; (3) sTBI + 5 mg/kg b.w. ILB^®^; (4) sTBI + 15 mg/kg b.w. ILB^®^. A fifth sham-operated control group also contained 12 rats with brains harvested for analysis at 2 days post-procedure, a time point that shows no significant difference in metabolic perturbation from that measured in sham-operated rat brains harvested at 7 days post-procedure (unpublished observations).

ILB^®^ was provided by Tikomed AB (Tikomed AB, Viken, Sweden) in 10 mL vials containing a solution of 20 mg/mL ILB^®^ (Tikomed AB, Viken, Sweden) in 9 mg/mL NaCl (Carlo Erba Reagents S.r.l., Cornaredo, Italy). A single batch of drug was used throughout the study (batch 3045586; expiry date 2019-02). ILB^®^ was diluted in sterile 9 mg/mL NaCl and injected subcutaneously (s.c.) in a volume of 0.5 mL in order to obtain a final administration of 1, 5, or 15 mg/kg b.w. of ILB^®^. The drug was administered in a single s.c. injection of 0.5 mL at 30 min after sTBI. The control group (*n* = 12) consisted of sham-operated animals that underwent the same procedure of anesthesia but received no sTBI or any s.c. injection and was sacrificed 2 days after the procedure.

### 2.2. Cerebral Tissue Processing for Biochemical Analyses

To minimize metabolite loss, an in vivo craniectomy was performed in all animals during terminal anesthesia. The rat skull cap was carefully removed, the brain was exposed, rapidly removed, the cerebral hemispheres dissected and placed on aluminium tongues pre-cooled in liquid nitrogen, freeze-clamped, and then immersed in liquid nitrogen. The freeze-clamping procedure was introduced to accelerate freezing of the tissue, thus minimizing potential metabolite loss [9,12,15,16]. Tissue homogenization for metabolite analyses was effected as described below. After the wet weight (w.w.) determination, the frozen hemispheres were placed into an ice-cold, nitrogen-saturated, precipitating solution (1:10 *w*/*v*) composed of HPLC-grade CH_3_CN (Carlo Erba Reagents S.r.l., Cornaredo, Italy)+ 10 mM KH_2_PO_4_ (Carlo Erba Reagents S.r.l., Cornaredo, Italy), pH 7.40, (3:1; *v*:*v*), and the homogenization was performed using an Ultra-Turrax homogenizer set at 24,000 rpm/min (Janke & Kunkel, Staufen, Germany). After centrifugation at 20,690× *g* for 10 min at 4 °C, the clear supernatants were saved, pellets were supplemented with an aliquot of 10 mM KH_2_PO_4_ and homogenized again as described above, and saved overnight at −20 °C in order to obtain a complete recovery of aqueous phase from tissue. A second centrifugation was performed (20,690× *g*, for 10 min at 4 °C) and the supernatants combined with those previously obtained. Clear protein-free samples were extracted by vigorous agitation with a double volume of HPLC-grade CHCl_3_ (Carlo Erba Reagents S.r.l., Cornaredo, Italy) and centrifuged as above. The upper aqueous phases (containing water-soluble low molecular weight compounds) were collected and subjected to chloroform washings for two more times (this procedure allowed the removal of all the organic solvent and of any lipid soluble compound from the buffered tissue extracts), adjusted in volumes with 10 mM KH_2_PO_4_, pH 7.40, to produce aqueous 10% tissue homogenates that were saved at −80 °C until assay.

### 2.3. HPLC Analysis of Energy Metabolites, Antioxidants, and Oxidative/Nitrosative Stress Biomarkers

Aliquots of each deproteinized aqueous brain sample were filtered through a 0.45 µm HV Millipore filter and loaded (20 µL) onto a Hypersil C-18, 250 × 4.6 mm, 5 µm particle size column, provided with its own guard column (ThermoFisher Scientific, Rodano, Milan, Italy) and connected to an HPLC apparatus consisting of a Surveyor System (ThermoFisher Scientific, Rodano, Milan, Italy) with a highly sensitive diode array detector (equipped with a 5 cm light path flow cell) set-up between 200 and 300 nm wavelength. Data acquisition and analysis were automatically performed by a linked PC using the ChromQuest 5.0^®^ software package provided by the HPLC manufacturer (ThermoFisher Scientific, Rodano, Milan, Italy).

The selected metabolites ATP, GTP, uridine triphosphate (UTP), cytidine triphosphate (CTP), ADP, AMP, hypoxanthine, xanthine, uric acid, inosine, guanosine, adenosine, NAA, NAD^+^, reduced nicotinamide adenine dinucleotide (NADH), oxidized nicotinamide adenine dinucleotide phosphate (NADP^+^), reduced nicotinamide adenine dinucleotide phosphate (NADPH), ascorbic acid, GSH, MDA, nitrite, and nitrate), related to tissue energy state and mitochondrial function, antioxidant defenses, and oxidative/nitrosative stress, were separated, in a single chromatographic run, according to slight modifications of existing ion-pairing HPLC methods formerly set up in our laboratory [20,21]. Assignment and calculations of the compounds of interest in chromatographic runs of tissue extracts were carried out at the proper wavelengths (206, 234, and 260 nm) by comparing retention times, absorption spectra and areas of peaks with those of peaks of chromatographic runs of freshly prepared ultrapure standard mixtures of known concentrations.

### 2.4. Statistical Analysis

Normal data distribution in each group was determined using the Kolmogorov–Smirnov test. Differences across groups were estimated by the one-way analysis of variance ANOVA. The Tukey’s Multiple Comparison Test was used as the post hoc test. Only two-tailed *p*-values of less than 0.05 were considered statistically significant.

## 3. Results

### 3.1. ILB^®^ Restores Cerebral Mitochondrial-Dependent Energy Metabolism and NAA Following sTBI

**Figure 1:**
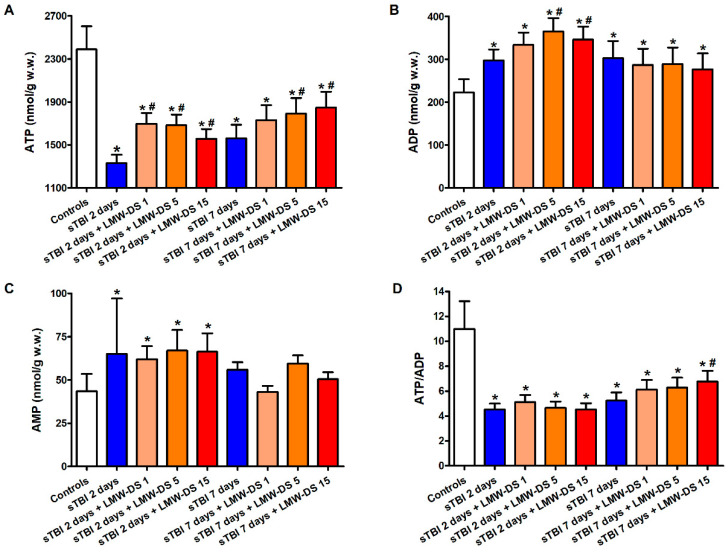
Effect of subcutaneous injection of increasing doses of LMW-DS (ILB^®^) on the cerebral concentrations of ATP (**A**), ADP (**B**), and AMP (**C**), and the ATP/ADP ratio (**D**), as a valuable indicator of the mitochondrial phosphorylating capacity. The parameters of interest were determined by high-performance liquid chromatography (HPLC) of deproteinized cerebral homogenates (at 2 and 7 days post-sTBI), both in untreated sTBI rats (TBI 2 days and TBI 7 days) and in rats receiving 1, 5, or 15 mg/kg b.w. LMW-DS (ILB^®^) administered 30 min after sTBI. Controls are represented by sham-operated rats. Values are the mean ± SD of 12 different animals and are expressed as nmol/g w.w. * Significantly different from sham-operated control rats, *p* < 0.05. # Significantly different from the corresponding time point of untreated sTBI rats, *p* < 0.05.

Concentrations of cerebral adenine nucleotides (ATP, ADP, and AMP) and the ATP/ADP ratio in sTBI-injured rats, in the absence or presence of increasing doses of ILB^®^ (1, 5, 15 mg//kg b.w.) are shown in Figure 1.

Compared to control sham-operated animals, sTBI produced a 44% and a 35% decrease in ATP concentration at 2 and 7 days post-injury, respectively (*p* < 0.05). This was accompanied by a significant increase in ADP and AMP, and a decrease in the ATP/ADP ratio at both time points (*p* < 0.05), indicating dysfunctional mitochondria as a cause of sustained imbalance of energy metabolism of cerebral cells. A single s.c. injection at 30 min post-injury of each of the three ILB^®^ doses positively affected brain energy metabolism. At 2 and 7 days after impact, cerebral ATP in ILB^®^-treated animals was significantly higher than the values measured in untreated sTBI rats (*p* < 0.05). Notwithstanding, ATP remained 23% lower than the value recorded in control rats (*p* < 0.05), even with the highest dose of ILB^®^. While ADP in ILB^®^-treated rats was significantly higher than the values of sham-operated controls at both 2 and 7 days after injury (*p* < 0.05), AMP measured at 7 days in ILB^®^-treated animals was not different from the AMP concentrations detected in the sham-operated controls. Consequently, the ATP/ADP ratio in ILB^®^-treated sTBI animals, measuring the mitochondrial phosphorylating capacity [41], was similar to that of untreated sTBI rats at 2 days after sTBI, but significantly increased at the highest dose tested at 7 days after injury (*p* < 0.05). At this time point, the ATP/ADP ratio was, however, still lower than the values measured in the sham-operated control rats (*p* < 0.05).

**Figure 2:**
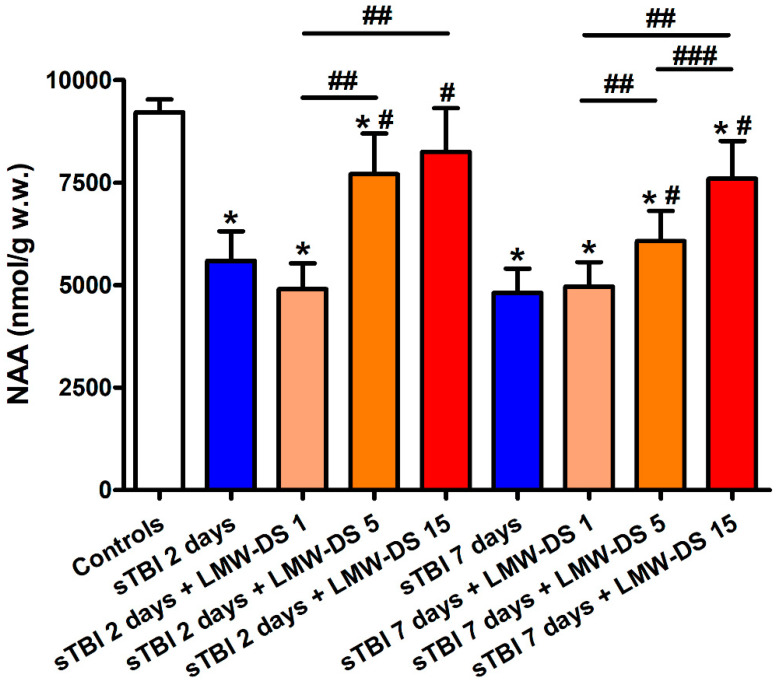
Effect of subcutaneous injection of increasing doses of LMW-DS (ILB^®^) on the cerebral concentrations of NAA, as a valuable indirect indicator of energy wellness and mitochondrial function. NAA was determined by HPLC on deproteinized cerebral homogenates at 2 and 7 days post-impact, both in untreated sTBI rats (TBI 2 days and TBI 7 days) and in rats receiving 1, 5, or 15 mg/kg b.w. LMW-DS (ILB^®^) administered 30 min after sTBI. Controls are represented by sham-operated rats. Values are the mean ± SD of 12 different animals and are expressed as nmol/g w.w. * Significantly different from sham-operated controls rats, *p* < 0.05. # Significantly different from the corresponding time point of untreated sTBI rats, *p* < 0.05. ## Significantly different from LMW-DS 1, *p* < 0.05. ### Significantly different from LMW-DS 5, *p* < 0.05.

The concentration of cerebral NAA in untreated sTBI animals (Figure 2) was decreased dramatically at both times post-impact (−39% at 2 days and −48% at 7 days, *p* < 0.05 compared to the value of controls), further evidencing the steady impairment of mitochondrial functions involving multiple biochemical processes of brain metabolism. Treatment with ILB^®^ at 30 min post-sTBI positively affected cerebral NAA levels, only when administered at 5 and 15 mg/kg b.w. Both doses attenuated the NAA decrease when measured at both 2 and 7 days after injury (*p* < 0.05 compared to levels at the corresponding times of untreated sTBI rats), even though these values remained significantly lower than the values measured in sham-operated control animals (*p* < 0.05). At 7 days post-injury, a dose-dependent effect of ILB^®^ administration was observed.

### 3.2. ILB^®^ Improves Cerebral High Energy Phosphate Concentrations and Reduces ATP Catabolism after sTBI

**Figure 3:**
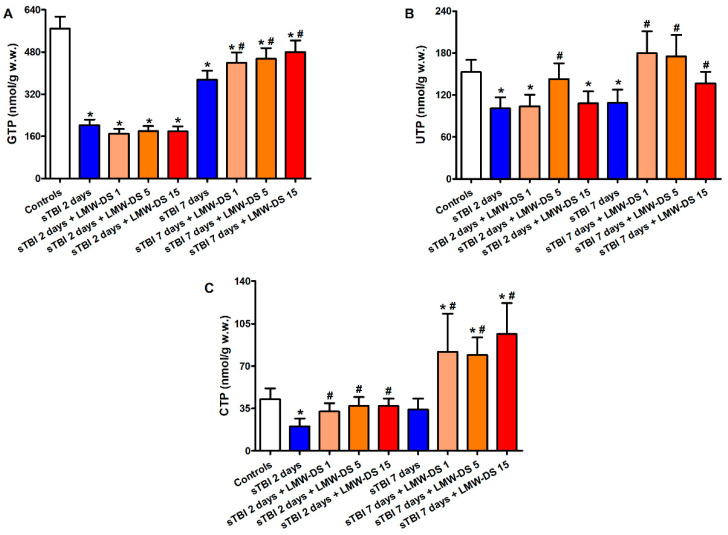
Effect of subcutaneous injection of increasing doses of LMW-DS (ILB^®^) on the cerebral concentrations of GTP (**A**), UTP (**B**), and CTP (**C**), as valuable indicators of the nervous cell energy state. The parameters of interest were determined by HPLC on deproteinized cerebral homogenates at 2 and 7 days post-impact, both in untreated sTBI rats (TBI 2 days and TBI 7 days) and in rats receiving 1, 5, or 15 mg/kg b.w. LMW-DS (ILB^®^) administered 30 min after sTBI. Controls are represented by sham-operated rats. Values are the mean ± SD of 12 different animals and are expressed as nmol/g w.w. * Significantly different from sham-operated control rats, *p* < 0.05. # Significantly different from the corresponding time point of untreated sTBI rats, *p* < 0.05.

Results of the cerebral triphosphate nucleosides GTP, UTP, and CTP measurements are illustrated in Figure 3.

At 2 and 7 days post-injury, the brains of untreated sTBI rats showed significant depletions in all of the three high energy phosphates (*p* < 0.05), with GTP being the compound most evidently affected by the insult (2.8 and 1.5 times lower than the values of controls at 2 and 7 days post-injury, respectively). The administration of all tested ILB^®^ doses produced beneficial effects, particularly evident at 7 days post-sTBI. At this time point, while GTP concentrations were still significantly lower than the values of sham-operated control animals (*p* < 0.05), levels of cerebral UTP were higher than those of untreated sTBI rats (*p* < 0.05) and not different from those of sham-operated control rats. Unexpectedly, brain CTP levels in animals receiving ILB^®^ were higher than those measured in both untreated sTBI and sham-operated control rats (*p* < 0.05).

**Figure 4:**
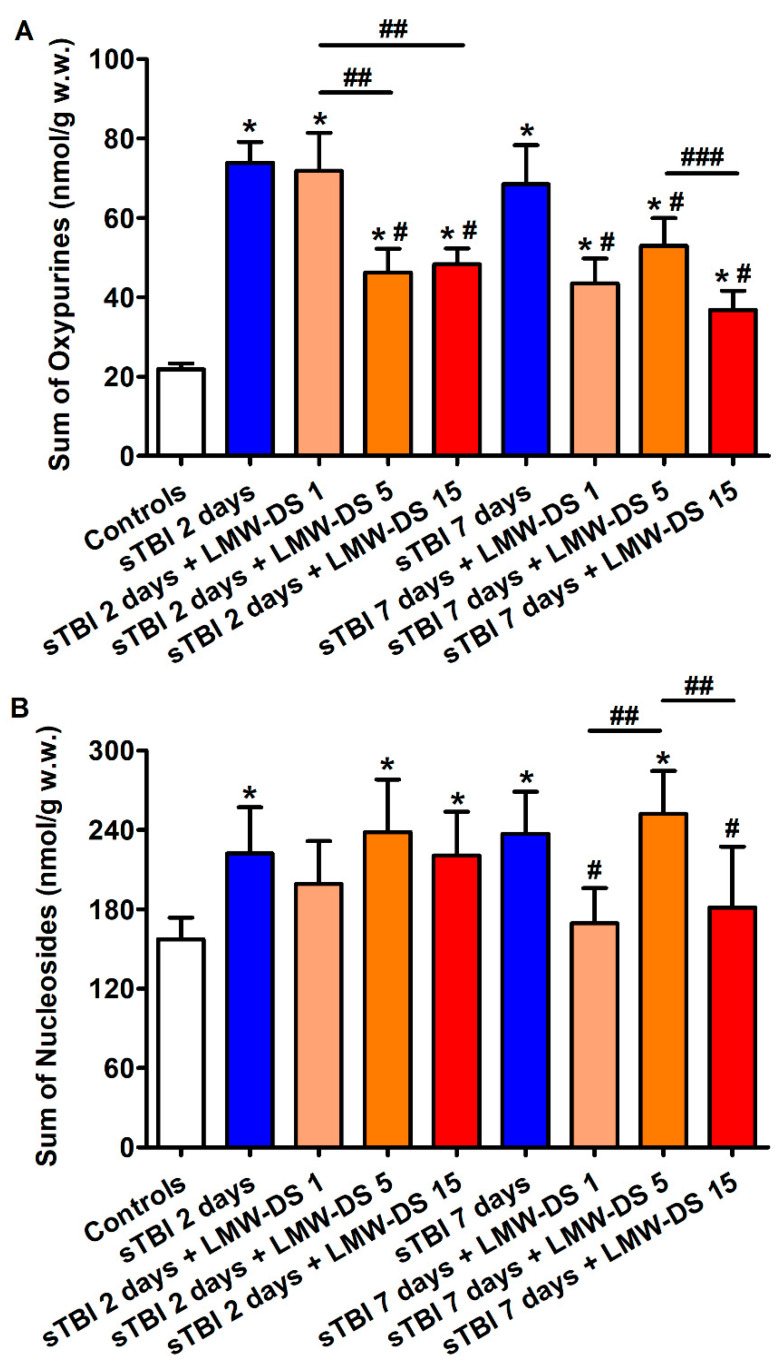
Effect of subcutaneous injection of increasing doses of LMW-DS (ILB^®^) on the cerebral concentrations of oxypurines (hypoxanthine + xanthine + uric acid) (**A**) and nucleosides (inosine + guanosine + adenosine) (**B**), as indicators of intracellular ATP catabolism in the brain. The parameters of interest were determined by HPLC on deproteinized cerebral homogenates at 2 and 7 days post-impact, both in untreated sTBI rats (TBI 2 days and TBI 7 days) and in rats receiving 1, 5, or 15 mg/kg b.w. LMW-DS (ILB^®^) administered 30 min after sTBI. Controls are represented by sham-operated rats. Values are the mean ± SD of 12 different animals and are expressed as nmol/g w.w. * Significantly different from sham-operated control rats, *p* < 0.05. # Significantly different from the corresponding time point of untreated sTBI rats, *p* < 0.05. ## Significantly different from LMW-DS 1, *p* < 0.05. ### Significantly different from LMW-DS 5, *p* < 0.05.

Concentrations of oxypurines (hypoxanthine + xanthine + uric acid) and nucleosides (inosine + guanosine + adenosine), deriving from purine nucleotide dephosphorylation, were significantly higher in the brains of untreated sTBI rats at both times post-injury compared to the values of sham-operated controls (*p* < 0.05), suggesting sustained imbalance in energy metabolism even at longer times after sTBI (Figure 4).

The sTBI-related increase in cerebral oxypurines and nucleosides was partly counteracted by ILB^®^ injection, with the effect more evident at 7 than at 2 days post-impact. In particular, at this time point oxypurine levels were significantly lower than those of untreated sTBI rats (*p* < 0.05), but still higher than the values of sham-operated controls (*p* < 0.05), while cerebral nucleoside concentrations were lower than those of untreated sTBI rats (*p* < 0.05) and not significantly different from the values of sham-operated animals.

### 3.3. Redox Metabolism of Cerebral Nicotinic Coenzymes after sTBI Is Improved by ILB^®^

**Figure 5:**
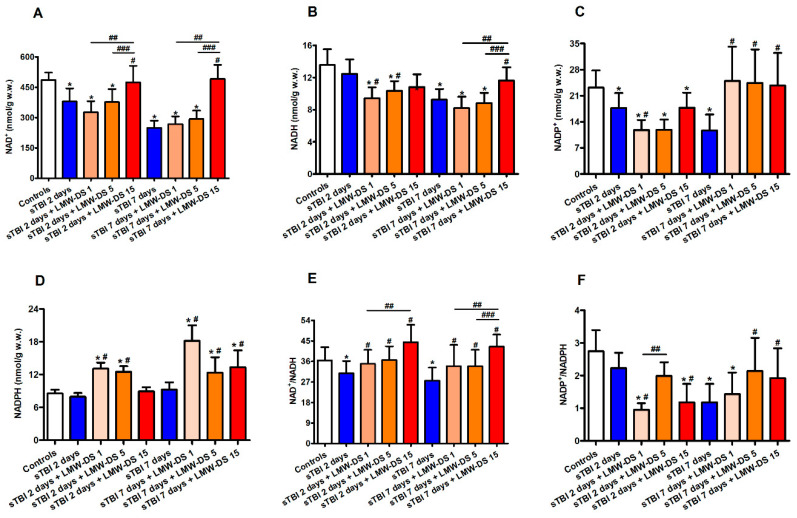
Effect of subcutaneous injection of increasing doses of LMW-DS (ILB^®^) on the cerebral concentrations of NAD^+^ (**A**), NADH (**B**), NADP^+^ (**C**), and NADPH (**D**), and of the NAD^+^/NADH (**E**) and NADP^+^/NADPH (**F**) ratios as valuable indicators of oxido-reductive metabolism of the nervous cell energy state. The parameters of interest were determined by HPLC on deproteinized cerebral homogenates at 2 and 7 days post-impact, both in untreated sTBI rats (TBI 2 days and TBI 7 days) and in rats receiving 1, 5, or 15 mg/kg b.w. LMW-DS (ILB^®^) administered 30 min after sTBI. Controls are represented by sham-operated rats. Values are the mean ± SD of 12 different animals and are expressed as nmol/g w.w. * Significantly different from sham-operated control rats, *p* < 0.05. # Significantly different from the corresponding time point of untreated sTBI rats, *p* < 0.05. ## Significantly different from LMW-DS 1, *p* < 0.05. ### Significantly different from LMW-DS 5, *p* < 0.05.

As already observed in previous studies (12), sTBI caused a progressive time-dependent depletion of oxidized (NAD^+^ and NADP^+^) and reduced (NADH and NADPH) nicotinic coenzymes, which is particularly evident at 7 days post-injury (Figure 5).

At this latter time point, the total pool of cerebral nicotinic coenzymes (NAD^+^ + NADH + NADP^+^ + NADPH) was 1.89 times lower (280.96 ± 36.75 nmol/g w.w.) than the value calculated in sham-operated controls (530.99 ± 39.52 nmol/g w.w., *p* < 0.05). Of relevance, the NAD^+^/NADH ratio was significantly lower at both time points after sTBI (−15% and −25%, respectively, at 2 and 7 days post-injury, *p* < 0.05 compared to sham-operated controls) suggesting an increased glycolytic rate during mitochondrial dysfunction. The beneficial effect of ILB^®^ administration was particularly evident, both at 2 and 7 days post-injury, with the highest dose tested (15 mg/kg b.w.) and involved each of the four nicotinic coenzymes (Figure 4). At this dosage, the total pool of cerebral nicotinic coenzymes (NAD^+^ + NADH + NADP^+^ + NADPH) was 512.83 ± 84.30 and 540.24 ± 73.70 nmol/g w.w. at 2 and 7 days after sTBI, respectively, i.e., equal to the value in the sham-operated control group and significantly higher than that in untreated sTBI rats (*p* < 0.05). It is worth underlining that the NAD^+^/NADH ratio increased in the brains of all three groups of ILB^®^-treated animals when measured at both time points following sTBI, in comparison with the values measured at the corresponding times in traumatized untreated rats (*p* < 0.05). At 7 days, the highest dose of ILB^®^ had significantly higher values than those calculated in rats receiving 1 or 5 mg/kg b.w. ILB^®^ (*p* < 0.05). The effect of on the NAD^+^/NADH ratio suggests a remarkable drug-induced tendency to decrease glycolysis and to recover mitochondrial metabolic oxidative processes finalized to energy production, namely, through the tricarboxylic acid cycle (TCA cycle), the electron transfer chain (ETC), and oxidative phosphorylation (OXPHOS).

### 3.4. ILB^®^ Ameliorates Changes in Cerebral Water-Soluble Antioxidants and Decreases Oxidative/Nitrosative Stress after sTBI

**Figure 6:**
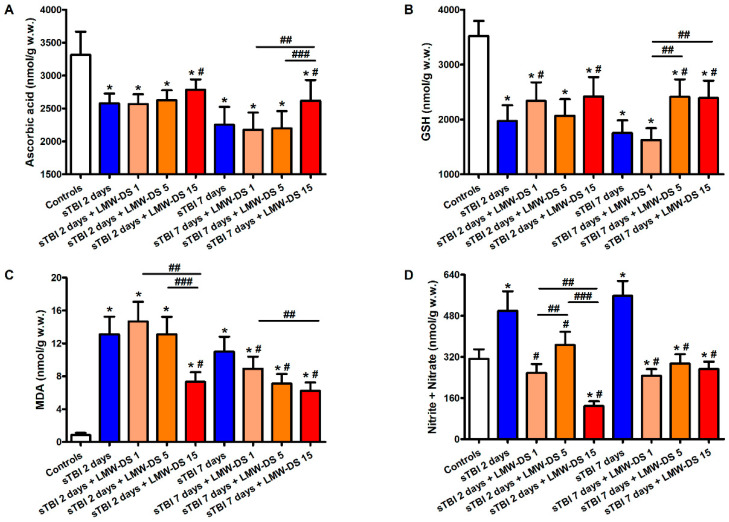
Effect of subcutaneous injection of increasing doses of LMW-DS (ILB^®^) on the cerebral concentration of the water-soluble low molecular weight antioxidants ascorbate (**A**) and GSH (**B**) and also of the biomarkers of oxidative/nitrosative stress, MDA (**C**) and nitrite + nitrate (**D**), which are valuable indicators of the nervous cell antioxidant defenses and ROS- and RNS-mediated damage. The parameters of interest were determined by HPLC on deproteinized cerebral homogenates at 2 and 7 days post-impact, both in untreated sTBI rats (TBI 2 days and TBI 7 days) and in rats receiving 1, 5, or 15 mg/kg b.w. LMW-DS (ILB^®^) administered 30 min after sTBI. Controls are represented by sham-operated rats. Values are the mean ± SD of 12 different animals and are expressed as nmol/g w.w. * Significantly different from sham-operated control rats, *p* < 0.05. # Significantly different from the corresponding time point of untreated sTBI rats, *p* < 0.05. ## Significantly different from LMW-DS 1, *p* < 0.05. ### Significantly different from LMW-DS 5, *p* < 0.05.

Animals receiving sTBI displayed a drastic depletion of the main water-soluble brain antioxidants, ascorbate, and GSH (Figure 6).

At 2 and 7 days post-injury, levels of cerebral ascorbate in untreated sTBI rats decreased by 22% and 32%, respectively, compared to the values measured in the brains of sham-operated controls. Similarly, the concentration of GSH, the main cellular reducing compound of the free -SH groups in proteins, underwent a 44% and 50% decrease at 2 and 7 days post-impact, respectively (*p* < 0.05 compared to the values in the sham-operated controls). The decrease in antioxidant defenses was accompanied by an increase in the biomarkers representative of oxidative/nitrosative stress, with MDA increasing 12- and 8-fold and the sum of nitrite + nitrate increasing 1.6- and 1.7-fold, at 2 and 7 days post-injury, respectively (*p* < 0.05 compared to the corresponding values detected in sham-operated controls). The treatment of sTBI rats with ILB^®^ was effective to protect cerebral levels of both of the water-soluble brain antioxidants, but only when administered at the highest dose tested (15 mg/kg b.w.). At this dosage, ILB^®^-treated rats had higher values of ascorbate and GSH concentrations than those measured in untreated sTBI animals, at both time points post-impact (*p* < 0.05). However, cerebral concentrations of both ascorbate and GSH remained significantly lower than the values in sham-operated animals (*p* < 0.05). ILB^®^ treatment decreased the extent of oxidative/nitrosative stress in the injured brain, particularly at 7 days after trauma, when cerebral levels of MDA and the sum of nitrite + nitrate were still higher than the corresponding values seen in the brains of sham-operated control rats (*p* < 0.05), but were significantly lower than concentrations measured in equivalent untreated sTBI rats (*p* < 0.05).

## 4. Discussion

The complex pathobiological mechanisms, the individual variability, the myriad of different causes of head injuries, and the consequent multitude of variables in the forces acting on the brain tissue at the time of impact render TBI as a difficult cerebral pathology to treat, with effective pharmacotherapies essentially absent [42,43]. For these reasons, the results from preclinical studies exploring efficacy and mechanisms of action of new potential treatments for TBI are of great interest.

The weight-drop closed-head impact acceleration model, set up by Marmarou et al. in 1994 [40], was used to induce reproducible brain damage characterized, at the biochemical, metabolic, molecular, and gene levels, in previous studies from our laboratories [12,22,23,24,25,26,29,30]. In the present study, we showed that a single post-injury administration of ILB^®^, a novel LMW-DS (5–8 kDa), decreased the damaging metabolic and oxidative/nitrosative stress induced in the rat brain by experimental sTBI, with a tendency to manifest dose-dependent effects on various metabolic parameters.

In the current experiments, we confirmed previous observations that cerebral metabolism in untreated sTBI rats underwent profound changes, particularly involving the capacity of an adequate supply of high-energy phosphates for nervous cell energy requirements. Compared to sham-operated controls, a decrease in cerebral levels of ATP, GTP, UTP, and CTP, and, most importantly, in the ATP/ADP ratio, were evident at both observational post-injury time points (2 and 7 days). The metabolic index (ATP/ADP ratio) is particularly relevant to metabolic function since it represents a valid index to evaluate the correct functioning of mitochondria through assessment of their main biochemical role, i.e., the coupling of the ETC to OXPHOS that ensures adequate ATP production for cell energy demand [41,44,45]. The imbalance between production and consumption of ATP leads to a remarkable increase in the level of its dephosphorylated products (oxypurines and nucleosides) measurable at both 2 and 7 days post-sTBI. The general metabolic derangement in brain tissue of untreated sTBI rats was also evidenced by the decreased concentrations of nicotinic coenzymes (compromising the efficiency of oxido-reductive reactions) and by the changes in their oxidized/reduced ratios. In particular, the decrease in the NAD^+^/NADH ratio corroborates the evidence of altered mitochondrial oxidative metabolism and indicates a switch to glycolytic-based metabolism for ATP supply in the post-injured brain. Dysfunctional mitochondria leading to energy failure are certainly related to the progressive decrease in NAA concentrations observed at 2 and 7 days post-sTBI, thus confirming previous observations demonstrating a strict connection between NAA homeostasis and mitochondrial function [29,30,44,45]. The altered ability to manage the tetravalent reduction of molecular oxygen to H_2_O in the ETC of the sTBI brain is probably one of the major determinants of sustained oxidative/nitrosative stress, generating the striking increase in cerebral levels of MDA and nitrite + nitrate, with the concomitant decrease in levels of the main water-soluble low molecular weight antioxidants (ascorbate and GSH).

In an attempt to counteract the aforementioned damaging effects of dysregulated brain metabolism in this study, we administered one single s.c. injection of increasing doses of ILB^®^ (1, 5, and 15 mg/kg b.w.) at 30 min after the induction of sTBI. The beneficial effects of ILB^®^ on mitochondrial-related energy metabolism in the post-injured brain were evident due to higher cerebral concentrations, not only of ATP and of the ATP/ADP ratio, but also of the triphosphate nucleosides (GTP, UTP, and CTP). This suggests that ILB^®^ administration improved mitochondrial function after sTBI, compared to that within the brains of untreated sTBI rats, permitting a general restoration of necessary brain energy metabolites required for a plethora of repair/biosynthetic cell processes [46,47]. The raised NAA concentrations found in the post-injured brain of ILB^®^-treated rats may have been due to acetyl-CoA, generated by the enhanced activity of the pyruvate dehydrogenase (PDH) complex, thereby regenerating NAA via the NAT8L-dependent biosynthetic reaction [48,49]. Recently, we found that, following sTBI, the genes controlling the sophisticated enzymatic mechanism regulating the PDH complex activity are expressed in an inhibitory mode causing an overall inhibition of the PDH complex and compromising restoration of energy metabolism of the post-injured brain [50]. Therefore, ILB^®^ treatment may disinhibit the PDH complex and potentiate acetyl-CoA production at an adequate rate to supply the TCA cycle, as well as to allow NAT8L-mediated condensation of acetyl-CoA with aspartate to form NAA. However, it should also be considered that higher values of NAA in ILB^®^-treated animals might have been due also to higher neuron integrity compared to that of untreated sTBI rats.

The improved mitochondrial-dependent cerebral metabolism of sTBI rats receiving ILB^®^ at 30 min post-impact supports the maintenance of higher levels of nicotinic coenzymes in treated brains compared to levels measured in untreated sTBI animals. The ILB^®^-induced increase in availability of NAD^+^ and NADH ensures glycolytic flux (via the NAD^+^-dependent glyceraldehyde-3-phosphate dehydrogenase reaction), PDH complex activity (via the NAD^+^-dependent acetyl-CoA formation), TCA cycle functioning (via the NAD^+^-dependent isocitrate dehydrogenase, α-ketoglutarate dehydrogenase, and malate dehydrogenase reactions), and enhanced electron flow through the ETC [51,52]. In addition, the increased availability of NADP^+^ and NADPH allows the tissue to efficiently perform pentose phosphate pathway and biosynthetic reactions (fatty acid biosynthesis for myelin regeneration), and also to catalyze important reductive reactions involved in cell mechanisms of defense (NADPH-dependent GSH-reductase reaction) [53,54]. This last important link was evidenced by the higher values of cerebral GSH measured in ILB^®^-treated animals, compared to those found in untreated sTBI rats. Higher cerebral GSH levels coupled to an increase in ascorbate, allowed animals treated with ILB^®^ to undergo lower oxidative/nitrosative stress-mediated tissue damage induced by sTBI, as indicated by the lower concentrations of MDA and nitrite + nitrate.

Dysfunctional mitochondria are considered as the main intracellular source of ROS [55,56]. However, additional contributors to oxidative stress, such as NADPH oxidase [57], activated microglia and macrophages during neuroinflammation [58], and an imbalance in iron metabolism [59], have all been shown to participate in ROS overproduction following TBI. RNS formation depends both on NO formation by the three isoforms of nitric oxide synthases (endothelial, eNOS; neuronal, nNOS; and inducible, iNOS) and by the concomitant overproduction of ROS [60]. It has also been shown that microglia, activated as part of the neuroinflammatory response to ROS formation [58], are largely responsible for most of the RNS production in the post-traumatized brain [61]. As the brain tissue of our untreated sTBI rats clearly showed biochemical signatures of mitochondrial dysfunction (decreased ATP/ADP ratio) and oxidative nitrosative stress (increased MDA and nitrite + nitrate), all the aforementioned sources of ROS and RNS production were likely contributing to the metabolic dysregulation during the post-injury period.

Post-sTBI administration of the sulfated polysaccharide ILB^®^ interfered with multiple targets, causing a significant decrease of mitochondrial dysfunction, energy derangement, and oxidative/nitrosative stress. Attenuation of oxidative/nitrosative stress may be the basis of the overall positive effect of ILB^®^ on brain metabolism. In fact, studies using various experimental models of cell toxicity have shown that different sulfated polysaccharides (SP) are able to attenuate oxidative stress by acting as efficient antioxidants [62], with scavenging properties directed towards superoxide anions and hydroxyl radicals [63] and some chelating capacity shown towards iron and, more evident, copper [64], as well as decreasing ROS formation thanks to their marked reducing potential [65]. Our results with ILB^®^ suggest that the concomitant decrease in lipid peroxidation (evidenced by the decrease in cerebral MDA) and NO production (decrease in nitrite + nitrate), and the increase in cell antioxidant defenses (increase in ascorbate and GSH) are mediated, at least in part, by the ability of ILB^®^ to act as an efficient ROS scavenger. Therefore, amelioration of mitochondrial activities (particularly TCA cycle, ETC, and OXPHOS) might be the result of the lower degree of oxidative/nitrosative stress. However, it should be underlined that previous studies performed with neuroblastoma cells in vitro and in the rodent brain after sTBI demonstrated that ILB^®^ dose-dependently modulates the gene expression and activation of multiple cytokines (e.g., IL1-β and TGF-β family) and growth factors (e.g., HGF, BDNF, and TNF), consistent with an anti-inflammatory phenotype (unpublished data). It is worth mentioning that Patel et al. [66] demonstrated the involvement of increasing levels of TGF-β1 in the stimulation of neuroinflammation, oxidative stress, and apoptosis in a model of rodent TBI, thus indicating that modulating the neuroinflammatory response following TBI has beneficial effects on multiple biochemical processes of the post-injured brain. It is also possible that, like some other dextran sulfates, ILB^®^ acts as a heparin mimetic, releasing and activating numerous heparin-binding growth factors sequestered in the extracellular matrix and prolonging their half-life in soluble form [67]. The release and activation of multiple growth factors from peripheral and central tissues would initiate a program of diverse downstream cellular responses relating to metabolism, tissue repair, and regeneration. For example, ILB^®^ induces release of Hepatocyte Growth Factor (HGF) from sequestered stores [35,68], a growth factor known to be neuroprotective and to stimulate glucose transport, metabolism and oxidative stress [69,70].

Previous studies, carried out in experimental models characterized by mitochondrial dysfunction, have also demonstrated that SP extracted from different biological sources had positive effects on mitochondrial membrane potential and apoptosis [71], as well as on the activity of the TCA cycle and ROS formation [72]. Interestingly, a recent study showed that the intraperitoneal administration of 1, 10, or 50 mg/kg b.w. of low molecular weight fucoidan (a sulfated polysaccharide extracted from brown algae) to rats before or after TBI induced by controlled cortical impact significantly attenuates mitochondrial dysfunction and brain oxidative stress [73]. This study also provided information on the therapeutic window for the SP administration, showing that the maximal effects of the drug were observed when the i.p. SP injection occurred between 0 and 2 h from injury [73]. Our results confirmed the correctness of the time of s.c. ILB^®^ administration (30 min after sTBI) and demonstrated that significant amelioration of the consequent mitochondrial dysfunction, deranged energy metabolism, and oxidative/nitrosative stress were obtained using lower doses of the drug compared to those administered in the study of Wang et al. [73]. Although direct indications of BBB penetration by ILB^®^ is currently difficult to be demonstrated as there are no valid tests to assay this compound in tissue samples (unless using radiolabeled administration of the drug), it is also possible to speculate, from the coincident results of this and previous study [73], that an aliquot of administered ILB^®^ and SP penetrated the blood–brain barrier (BBB), as BBB integrity is known to be disrupted after TBI, allowing BBB penetration by compounds like ILB^®^ characterized by low molecular mass [74]. Notwithstanding, Barzò et al. found restoration of BBB functions after 30 min from sTBI [75]; it is worth underlining that studies using the same [76] or similar closed impact models of sTBI [77,78], and evaluating BBB dysfunction for longer times post-impact, showed altered BBB permeability for up to 48 h post-injury. Furthermore, evidence suggests that molecules up to 70 kDa cross an opened blood–brain barrier, with smaller molecular weight compounds passing more freely [79]. Therefore, it seems quite reasonable to assume that dysfunctional BBB occurred even in our sTBI rats allowing a facilitating access of aliquots of the drug to the brain tissue in those animals receiving ILB^®^ administration 30 min after injury.

## 5. Conclusions

In conclusion, the present study provides evidence that the therapeutic administration of ILB^®^ after sTBI dose-dependently improves brain metabolism, inhibits oxidative/nitrosative stress, and avoids the depletion of low molecular weight antioxidants. Particularly relevant is the restoration of mitochondrial function, allowing resolution of the sTBI-induced energy crisis with rescuing of the ATP/ADP ratio and triphosphate nucleotide concentrations. Although these results do not establish whether ILB^®^ effects are due to a direct influence on mitochondrial activity (secondarily, the improved mitochondrial functions led to a decrease of ROS and RNS production, oxidative/nitrosative stress, and neuroinflammation), to a direct powerful antioxidant capacity (secondarily, the decrease of ROS and RNS production led to improved mitochondrial functions, oxidative/nitrosative stress inhibition, and reduction of neuroinflammation), or to a neuroimmunomodulator activity (secondarily, the decrease of neuroinflammation led to improved mitochondrial functions, decrease of ROS and RNS production, and oxidative/nitrosative stress inhibition), it is possible to affirm that ILB^®^ represents a promising therapeutic agent to decrease the tissue damage associated with sTBI. Further studies to better characterize ILB^®^ effects and mechanisms of action are in progress.
